# Quality Parameters and Consumer Acceptance of Jelly Candies Based on Pomegranate Juice “*Mollar de Elche*”

**DOI:** 10.3390/foods9040516

**Published:** 2020-04-20

**Authors:** Marina Cano-Lamadrid, Ángel Calín-Sánchez, Jesús Clemente-Villalba, Francisca Hernández, Ángel A. Carbonell-Barrachina, Esther Sendra, Aneta Wojdyło

**Affiliations:** 1 Research Group “Food Quality and Safety”, Department of Agro-Food Technology, Escuela Politécnica Superior de Orihuela (EPSO), Universidad Miguel Hernández de Elche (UMH), Ctra. Beniel, km 3.2, 03312 Orihuela, Alicante, Spain; marina.cano.umh@gmail.com (M.C.-L.); acalin@umh.es (Á.C.-S.); jesus.clemente@hotmail.com (J.C.-V.); angel.carbonell@umh.es (Á.A.C.-B.); esther.sendra@umh.es (E.S.); 2 Research Group “Plant Production and Technology”, Department of Plant Sciences and Microbiology, Escuela Politécnica Superior de Orihuela (EPSO), Universidad Miguel Hernández de Elche (UMH), Ctra. Beniel, km 3.2, 03312 Orihuela, Alicante, Spain; francisca.hernandez@umh.es; 3 Department of Fruit, Vegetable and Plant Nutraceutical Technology, Wrocław University of Environmental and Life Sciences, 37 Chełmońskiego Street, 51-630 Wrocław, Poland

**Keywords:** *Punica granatum*, *Malus domestica*, honey, sensory analysis, descriptive, PDO

## Abstract

There is an upward trend towards reducing or suppressing additives in foods, as well as reducing the use of E-numbers in labels providing clean label foods. Therefore, the development of confectionary products based exclusively on natural ingredients with antioxidant properties may offer valuable solutions to the confectionery industry. Fruit juices and purées may provide functional and organoleptic properties in jelly candies in a natural way. The consumption of pomegranate fruit and derivative products has increased due to their association with health benefits. The aim of this study was to determine consumer insights about pomegranate-based jellies, cultivar “*Mollar de Elche*”, as affected by formulation (100% pomegranate juice or 50%–50% pomegranate juice–apple purée) and type of sweetener (sugar or honey), and to link affective and descriptive data from sensory studies. The most valued quality parameter of pomegranate products, red color (measured by the green–red coordinate, a*), was not negatively affected by jelly preparation. It was determined that the main liking drivers for pomegranate jellies were intense red color and high brightness. The results might be used by pomegranate processing companies to improve their manufacturing protocols and to develop successful products meeting consumer demands and needs. The formulation containing 20% gelatin, pure “*Mollar de Elche*” pomegranate juice, 1% citric acid, and sucrose as sweetener provided the best quality of jellies in terms of color, texture, antioxidant capacity, and sensory attributes.

## 1. Introduction

Gummies consist of food products whose main ingredient is sugar, normally incorporated in the form of sucrose syrup and/or glucose, combined with gelling agents, acids, aromas, and food colorants [1]. Within this category, two confections can be differenced according to the type of gelling agent used: (i) jellies and (ii) gummies, using gelatin and other hydrocolloids such as pectin, respectively [2]. Due to the high contents of sugar and food additives, as well as the nondesirable compounds generated by the heat treatment, it has been reported that a high consumption of such confections may have negative effects on human health [3,4,5]. In spite of this knowledge, both jellies and gummies are consumed by a large and heterogeneous group of people from children to elderly persons [3,6].

According to recent studies, it has been reported that (i) most of the Spanish population consume more sugar than recommended [7], (ii) adult American males and females ingest up to 150 and 100 kcal of added sugars per day, respectively [8], and (iii) 56% of United Arab Emirates students were considered heavy sugar consumers [9]. The high consumption of sugar in children has been linked with obesity, impulsiveness, addictive behavior, and stress-driven anxiety [10,11]. Recently, it has been found that honey-fed rats showed significantly less anxiety throughout the study as compared with those fed with sucrose [11]. Taking this into account, both a reduction of sugar content and the replacement of sugar with other alternatives [12,13] such as honey could be healthier alternatives for gummies and jellies [14]. The use of healthier alternatives such as orange, strawberry, and black mulberry juices as well as honey has been considered for the manufacturing of jellies [5].

Additives (color agents, flavorings, etc.) have been considered as potential carcinogens and/or neurotoxic agents by themselves or by the contaminants they contain [15]. Although these compounds can be used due to their safety, there is an upward trend towards their suppression and a high demand for clean label foods, without food additives or E-additives. Therefore, the development of confectionary products based on natural ingredients with antioxidant properties is a current trend to obtain new and healthier products [3]. Because gummies and jellies are widely consumed, they can be considered a good vehicle to increase the intake of functional substances such as fiber and phenolic compounds [3,6]. Fruit juices and purées are the current alternative by the food industry to improve the organoleptic properties of gummies and jellies in a natural way (color, flavor, and texture), and even fruit by-products have been used [2,3,6].

Pomegranates (*Punica granatum* L.) are not only consumed fresh but are also used in the preparation of industrialized foods, such as jams, fermented milks, juices, smoothies, jellies, wines, and dried snacks [16,17,18,19]. During the last decades, pomegranate consumption has increased due to its benefits on human health [20,21]. It has been demonstrated that pomegranate presents a low caloric index and its composition highlights the presence of citric acid, polyphenols, and vitamin C. Apart from these functional compounds, pomegranate juice presents techno-functional properties, being useful, for example, as a natural colorant. Additionally, fruit purée or fruit pomace with high pectin content, such as that from apples, has been reported as suitable to obtain jellies and gummies with a smooth texture [22,23]. A previous study described the production of a reduced-sugar pomegranate juice jelly supplemented with an aqueous extract of pomegranate by-product (peel) [24].

Considering all the above, the aim of this study was to determine consumer insights about pomegranate-based jellies (cultivar “*Mollar de Elche*”) as affected by formulation (pure pomegranate juice or 50%–50% pomegranate juice–apple purée) and type of sweetener (sugar or honey), and to link consumer data to descriptive sensory analysis. The results might be used by companies producing or selling pomegranate coproducts to improve their procedures and make their products more successful by meeting consumer demands and needs. In addition, the following parameters were studied to make the correct decision: (i) physical parameters (color), (ii) mechanical properties (texture), (iii) antioxidant capacity (DPPH, FRAP, and ABTS^+^), and (iv) affective and descriptive sensory analyses.

## 2. Materials and Methods

### 2.1. Experimental Design

The research consisted of two parts: 

In the first part of the study, the effects of three gelatin doses (15%, 17%, and 20%), two sweeteners (sucrose or honey), and two juice–purée ratios (100–0 and 50–50) on properties of pomegranate jelly candy were investigated. The parameters controlled were antioxidant capacity, color, and texture. Additionally, the effect of the addition of citric acid (1%) was evaluated on color and antioxidant capacity. 

In the second part of the study, a sensory evaluation was carried out using only confections prepared using the selected gelatin dose (20%) and with citric acid addition (1%) but still evaluating the effect of two sweeteners (sucrose or honey) and two juice–purée ratios (100–0; 50–50).

With this design, the number of formulations in the first stage was 12 (n = 3 × 2 × 2) while in the second part of the study, the number was 4 (n = 2 × 2). Three jelly candies for each formulation were obtained as replicates for mechanical analysis, color coordinates, and antioxidant capacity.

### 2.2. Chemicals

The acetic acid, hydrochloric acid, Trolox (6-hydroxy-2,5,7,8-tetramethylchroman-2-carboxylic acid), 2,2′-azinobis-(3-ethylbenzthiazoline-6-sulfonic acid) (ABTS), 2,2′-azobis (2-amidino-propane) dihydrochloride (AAPH), potassium persulfate, TPTZ (2,4,6-tripyridyl-1,3,5-triazine), and FeCl_3_ were purchased from Sigma-Aldrich (Steinheim, Germany).

### 2.3. Plant Material and Jelly Candy Preparation

Pomegranate fruits, cultivar “*Mollar de Elche*”, were collected from a farm located in Elche, Alicante (Spain) with Protected Designation of Origin (PDO) and apple fruits, cultivar “*Smith*”, were obtained from a local supermarket. Pomegranate fruits were hand-harvested at a commercial maturity stage (14° Brix and 0.20% citric acid for “*Mollar de Elche*” pomegranates), whereas apples were characterized by having 8 °Brix and 0.75 % malic acid. Both types of fruits were stored at optimal conditions (8 °C and HR 90%–95%, and 1 °C and HR 90%–95%, respectively, for pomegranates and apples). 

The different stages of the fruit-based jelly candy preparation process were:

1. Purée preparation: apples were cut, ground, and heated at 80 °C in Thermomix device (Vorwerk, Wuppertal, Germany); 10 mL of citric acid per 1 kg of fruit was added to prevent enzymatic browning of the fruit [25]. Finally, the particle size of the mixture was reduced in a blender until a thin purée was obtained (apple purée, AP).
2. Pomegranate juice preparation: pomegranate fruits were cut in halves, arils were manually separated from the husk, and juices were prepared using only arils (pomegranate juice, PJ).
3. Gelatin hydration: gelatin was hydrated with water at 25 °C for 10 min (using ratios of 13%, 17%, and 20 %).
4. Heat treatment and homogenization: the final blend included 25% of sweetener (sucrose or honey) and 31%, 29%, and 27.5% of pomegranate juice for the product with 13%, 17%, and 20% of gelatin, respectively. These blends were heated at 60 °C for 4 min in a Thermomix device (Vorwerk, Wuppertal, Germany) and then the same percentage (31%, 29%, and 27.5%) of pomegranate juice or apple purée was added and mixed at 60 °C for 2 min. Finally, the different formulations were obtained (Table 1).

The molding and maturation step was carried out at 4 °C for 24 h.

### 2.4. Antioxidant Capacity (DPPH, FRAP, ABTS+) and Total Polyphenol Content

Methanol extract was prepared as follows: jelly candies (1 g) were mixed with 10 mL of MeOH/water (80:20, v/v) + 1% HCl, sonicated at 20 °C for 15 min, and left for 24 h at 4 °C. Then, the extract was again sonicated for 15 min (TSD-J 0.7 L, 50 W, 40 kHz) and centrifuged at 15,000× *g* for 10 min (Sigma 3–18 K, Osterode and Harz, Germany). The antioxidant capacity (AOC) and total polyphenol content (TPC) were determined using DPPH, ABTS^+^, and FRAP assays, and quantified using Folin–Ciocalteu reagent [26,27]. As to DPPH, 10 μL of the supernatant was mixed with 40 μL of MeOH and added to 950 μL of DPPH solution. The mixture was shaken and placed under dark conditions for 15 min. The absorbance was measured at 515 nm. Additionally, 10 μL of the supernatant was mixed with 990 μL of ABTS or FRAP solutions. After 10 min of reaction, the absorbance was measured at 734 nm for ABTS and 593 nm for FRAP. Determinations were performed using a UV-2401 PC spectrophotometer (Shimadzu, Kyoto, Japan). Calibration curves, in the range 0.5–5.0 mmol Trolox L^−1^, were used for the quantification of the three methods of antioxidant activity showing good linearity (*R*^2^ ≥ 0.998). Analyses were run in triplicate per each jelly candy replicate (n = 9) and results were expressed as mg/100 g fw (fresh weight).

### 2.5. Color Parameters

Color coordinates (L*, a*, and b*) were determined by reflectance measurement with a Color Quest XE Hunter Lab colorimeter (Illuminant D65 and 10° observer angle). Measurements were run in triplicate per each jelly candy replicate (n = 9).

### 2.6. Texture

A texture profile analysis (TPA) was performed using a texturometer (TA-XT2i, Stable Micro Systems, Surrey, England), following a previous method for jelly candies [5] with slight modifications. Instead of using a sphere probe, a P0.5R cylinder (AOAC reference for gelatin analysis) was used, and the test conditions were as follows: pretest speed 2 mm/s, test speed 1 mm/s, post-test speed 1 mm/s, waiting time between cycles 2 s, trigger force 5 g, and 75% compression. The parameters measured were the following: hardness (H), adhesiveness (A), springiness (S), cohesiveness (Co), gumminess (G), and chewiness (Ch). The texture profile analysis was performed within the same gelation container, executing four measurements per each jelly formulation replicate (n = 12). 

### 2.7. Descriptive Sensory Analysis

In order to run the sensory test, jellies were prepared in a teddy bear candy shape. Eight trained panelists from the *Escuela Politécnica Superior de Orihuela* (EPSO), University Miguel Hernández de Elche, UMH (aged 26 to 55 years old, four females and four males), and with more than 500 h of experience in sensory testing, participated in this study. The panel was selected and trained following the ISO standard 8586-1 (1993), and it is specialized in descriptive sensory evaluation of pomegranate products [28,29]. For the present study, the panel worked during two orientation sessions (90 min for each one) discussing the main organoleptic characteristics of pomegranate jelly. The lexicon used for describing the flavor and texture attributes was previously developed by other authors [30,31]. Both lexicons were adapted for jelly based on pomegranate during the orientation sessions. The methodology for serving samples and the scale used for quantifying the intensity of each evaluated attribute were those indicated in a previous study [28]. The pomegranate jellies used for the descriptive sensory analysis were prepared using nonstick molds which were flexible for easy removal with 50 teddy bear cavities (size: 19 × 14 cm).

### 2.8. Consumer Study

A consumer panel of 100 volunteers recruited from the SensoFood Solutions database evaluated the samples. At the beginning of the test, consumers were presented with a consent form with general information about the test and their willingness to participate in the study. Two experimental conditions were investigated: blind and informed. Participants were randomly divided into two groups of 50 people: one of the groups performed a blind evaluation of the samples, whereas the other performed an informed evaluation. Information on the exact formulation used in the preparation of each samples was provided to the consumers participating in this section of the study; this was (i) sample PJAPH-20c contains honey, pomegranate juice PDO “*Mollar de Elche*”, and apple; (ii) sample PJH-20c consists of honey and pomegranate juice PDO “*Mollar de Elche*”; (iii) PJAPS-20c: sugar, pomegranate juice PDO “*Mollar de Elche*”, and apple; and (iv) PJS-20c: sugar, pomegranate juice PDO “*Mollar de Elche*”. The consumer study was carried out in the official testing room of UMH and each consumer evaluated all four samples in a single session in a randomized way. Consumers were asked about their overall liking on a nine-point hedonic scale followed by questions about the appearance, flavor, and texture attributes. Additionally, a nine-point Likert scale was used for the Just About Right (JAR) questions to determine possible improvements of the attributes: overall liking, color, appearance, fruity-ID, pomegranate-ID, sweetness, sourness, hardness, solubility, and adhesiveness. 

### 2.9. Statistical Analysis

Multifactor analysis of variance (ANOVA) [factor I: formulation (pomegranate juice, or pomegranate juice and apple purée); factor II: gelatin percentage; factor III: sweetener (sugar or honey); and/or factor IV: information] was performed using StatGraphics Plus 5.0 software (Manugistics, Inc., Rockville, MD), followed by Tukey’s multiple range test. Partial least-square regression (PLS) analysis was carried out to study the relationship of the descriptive analysis (x: independent variables) with the overall liking data (y: dependent variable) using XLSTAT Premium 2016 (Addinsoft, Barcelona, Spain). Finally, penalty analysis was conducted to provide extra information about the possible improvements of some of the samples.

## 3. Results and Discussion

### 3.1. Percentage of Gelatin Selection 

The antioxidant capacity of jellies, assayed by 3 methods (ABTS^+^, DPPH, and FRAP), TPC, color coordinates, and several texture parameters were the parameters which determined the proper percentage of gelatin addition (Table 2). ABTS^+^ values ranged from 2.46 (PJAPS-20) up to 3.69 mg/100 g fw (PJAPS-13) with no statistically significant differences being found for formulation and sweetener; however, the percentage of gelatin caused significant differences. The highest values of ABTS^+^ were found for samples with the addition of 13% or 17% of gelatin. DPPH values varied from 10.6 to 14.9 mg/100 g fw for PJAPS-20 and PJS-20, respectively. In this case, the factor sweetener did not cause significant differences but the factors formulation and concentration of gelatin produced significant differences. FRAP analysis reported values ranging from 1.41 mg/100 g fw for the case of PJAPH-20 up to 4.59 mg/100 g fw in the case of PJS-13. Statistical analysis of FRAP antioxidant capacity values showed a similar trend to that of DPPH. TPC ranged from 72.0 mg/100 g fw up to 159 mg/100 g fw. It is well known that the antioxidant capacity is positively correlated with the polyphenol content, especially in the case of pomegranate fruit [32]. In general, it can be stated that the higher the gelatin content, the lower the functional properties of jelly candies, considering both the antioxidant capacity and the TPC [1]. On the other hand, there was a clear effect on the addition of honey instead of sucrose regarding the antioxidant capacity because honey has significantly higher antioxidant power [14].

The color analysis results determined that L*, a*, and b* parameters of jelly candies were significantly affected by the formulation, the gelatin dose, and the addition of sweetener (only a* and b*) (Table 2). The color of the jelly candies enriched with apple purée got darker during the hot mixing step; this may be as a result of nonenzymatic browning reactions, such as the Maillard reaction and caramelization at high temperatures, among other reasons. The Maillard reaction occurs among amino groups and reducing sugars, with high temperatures accelerating such reactions and resulting in changes in the aroma, taste, and especially color of foods [33]. Although a pretreatment was made to avoid enzymatic reaction by heating at 80 °C [25] and adding citric acid to the apple purée, it can be possible that it was not enough due to the high browning rate of Granny Smith as compared to other cultivars [34]. In addition, a previous study about pomegranate jelly obtained higher reddish color (a* values between 4.4 and7.6), due to type and percentage of gelling agent (guar, xanthan, and tragacanth gums at 1%), and probably a different cultivar of pomegranate juice which was not indicated [24].

The TPA test consists of compressing a food sample in a reciprocating motion that imitates the action of the jaw. Table 2 shows the results of several texture parameters (hardness: H; adhesiveness: A; springiness: S; cohesiveness: Co; gumminess: G; and chewiness: Ch). Regarding hardness, both formulation and gelatin percentage had significant effects, with the highest and lowest values being those of the samples PJH-20 and PJAPS-13, respectively. At this point, honey-sweetened jellies would be preferred because honey is healthier than sucrose and no significant effects on the type of sweetener were observed on their hardness [14]. The rest of texture parameters showed a particular behavior depending on the different variables. All three factors evaluated significantly affected the jellies’ adhesiveness, with the PJH-20 sample having an intermediate value which can be accepted as optimal; similar intermediate values were obtained for jellies prepared using 20% gelatin. Therefore, samples with this percentage of gelatin (20%) were selected for the next part of the experiments. It is worth mentioning that in future research, it may be of interest to test pectin as a gelling agent as it has been suggested in recent studies [35].

### 3.2. Addition of 1% Citric Acid

The addition of 1% citric acid to pomegranate jellies significantly affected the color coordinates (L*, a*, and b*) and the AOC (Table 3). Jellies prepared with 1% citric acid had higher values of L* (darker color) and also a* and b* (more intense red color) than those without acid addition. One of the most important quality characteristics of the pomegranate is the intense red (garnet) pigmentation of its arils, juices, and related products [17]. This red color depends on total anthocyanin content but also on the chemical structure of the individual anthocyanin [36]. It is known that the lower the pH of the juice, the more stable the anthocyanins [37]. This red/garnet color is highly attractive for consumers and its preservation during the jelly preparation is essential. The color behavior in this study is similar to those reported in other studies [28].

On the other hand, the response of the AOC values, evaluated using three methodologies (ABTS^+^, FRAP, and DPPH), to the citric acid addition showed different trends for each assay. In this way, the addition of 1% citric acid caused an increase in the DPPH values but a decrease in the FRAP values, as compared with control jellies, while no significant effect of citric acid addition was observed on ABTS^+^ values (Table 3). This disagreement is due to the fact that each method is specific for only one antioxidant mechanism, so they do not necessarily have to behave in the same way. In this way and according to previous studies [38], one of the major problems with the evaluation of the antioxidant activity of biological materials is the choice of the proper assay.

The addition of citric acid increased the TPC by 16% as compared to the control jellies. These results were similar to those previously reported by Kim and Padilla-Zakour [39], who reported values of 132.9 mg GAE 100 g^−1^ for cherry jelly and 144.3 mg GAE 100 g^−1^ for plum jam. In addition, the current findings agreed with a recent study [40], which observed that the processing of sapota pulp jelly showed an increase of 4.8 times from the fresh fruit to the processed fruit. Heat treatment can modify the content of phenolic compounds due to disruption of the plant cell wall, with the consequent release of these compounds [41].

It was found that the higher the DPPH values, the higher the TPC values. This was in accordance with recent studies [42,43], which reported this positive and significant relationship (*R*^2^ = 0.733 for different plant extracts and *R*^2^ = 0.899 for jellies with *Musa acuminata* Colla peels). The strong relationship between TPC and free radical scavenging activity might be due to the combined effect of various phenolic compounds and their high hydrogen atom donating abilities [44]. In summary, it can be concluded that citric acid addition at 1% improves the color, antioxidant activity, and TPC of the pomegranate jellies.

### 3.3. Descriptive Sensory Analysis, Consumer Acceptability, and Driving Sensory Attributes

The conditions rendering the best functional results (20% gelatin and 1% citric acid) were used to prepare the pomegranate jellies used for the descriptive and affective sensory studies. This study will provide us with information on the drivers of liking for pomegranate jelly candies and offer industry-relevant information regarding their formulation.

Descriptive sensory analysis was performed to assess the sensory profile of the pomegranate jelly candies and to check significant differences among formulations. The effect of formulation (the addition of pomegranate juice and/or apple purée) and sweetener (honey and sucrose) was studied, and 10 flavor and texture attributes were evaluated (Table 4). 

The highest color intensity (the higher the value, the more intense reddish the color) was observed when pomegranate juice and sucrose were used (PJS-20c), with lower values being found when honey was used. Previous studies reported that the addition of honey prevented browning reactions in preparation of several fruit juices and fruit-based products, but current results showed the opposite behavior [45]. The presence of amino acids in honey that can easily react with the sugars when heating begins breaking the molecular bonds in the honey (nonenzymatic reaction), thus changing the color of the jellies. Additionally, although citric acid was added to the apple purée to avoid enzymatic browning and Maillard browning, the addition of apple purée reduced color values and brightness. It has been demonstrated that the addition of apple purée increases brownish notes due to enzymatic browning, although it depends on the cultivar used [34]. The cultivar used (Granny Smith) could be substituted by other cultivars with less polyphenol oxidase activity such as Fuji or Golden [34]. The sweetness of these jellies was adjusted to low values because consumers are used to the high-sugar commercial confections (with at least 7% of sugar content). One of the purposes of this work was to reduce the sugar content. Apart from that, it is important to highlight that no differences in sweetness were found between sweeteners, but a nonsignificant increase was noticeable when apple purée was used. It is worth mentioning no off-flavor was detected and higher values of apple ID were observed as compared to those of pomegranate ID. Previous studies indicated that “*Mollar de Elche*” pomegranate juices presented apple notes [46]; this could be the reason for the observed trend. In addition, previous studies indicated that the use of apple juice in a pomegranate-based product led to samples with low color intensity and low pomegranate flavor but high intensity of apple; the apple flavor masks that of pomegranate [17].

Mean scores for liking of color, appearance, some flavor notes (fruity, pomegranate), basic taste (sweetness, sourness), texture attributes (hardness, solubility, and adhesiveness), and overall liking of samples are shown in Table 5. In general, all the tested jellies obtained scores above 5.0, on a nine-point hedonic scale, and consequently can be considered as acceptable, although their acceptance can be clearly improved. Besides, it is important that the effect of providing information about the nature of the jellies to consumers was also evaluated. Regarding the formulation factor, there were no statistical differences for both the overall liking and that of the rest of attributes. Regarding the sweetener factor, the use of sugar was the most liked one by consumers, presenting the highest scores for overall, color, appearance, and fruity ID liking. Making consumers aware of the nature of the jellies they were consuming made a difference, leading to higher scores for overall liking and the satisfaction degree for most of the evaluated attributes (appearance, pomegranate ID, sourness, hardness, solubility, and adhesiveness). A recent study found similar results, with the overall liking of pomegranate-based drinks increasing after information which was described previously in the Materials and Methods section was provided to the consumers [47].

**Figure 1:**
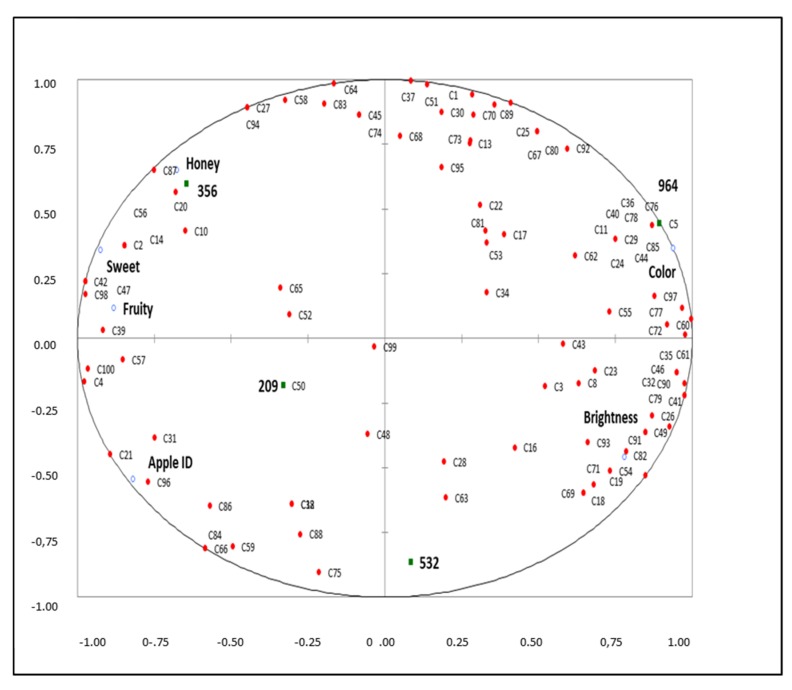
Partial least squares regression (PLS) of the descriptive sensory profile (X) and consumer overall liking (Y) of the samples (squares = samples; filled circle = consumers; unfilled circle = descriptive attributes).

Figure 1 represents a correlation mapping of consumer overall liking with the descriptive attributes (only statistically significant ones were used) to determine the drivers of liking. Two well-separated groups of consumers were observed. On the right side, the majority of consumers were grouped close to the attributes color and brightness. Current results agreed with previous studies, indicating that color of pomegranate-based products is one of the most important parameters [28]. On the opposite side, fewer consumers were grouped close to fruity, apple ID, honey, and sweetness attributes, indicating that a smaller possible cluster of consumers could prefer these sensory attributes. These results indicated that most consumers would choose samples which are characterized by reddish color (PJcS). Industry could use these liking drivers as quality indicators for improving their commercial products. It is worth mentioning that some expected sensory attributes as consumer drivers were not selected since the values generally were not sufficiently high in the present developed pomegranate jellies.

**Figure 2:**
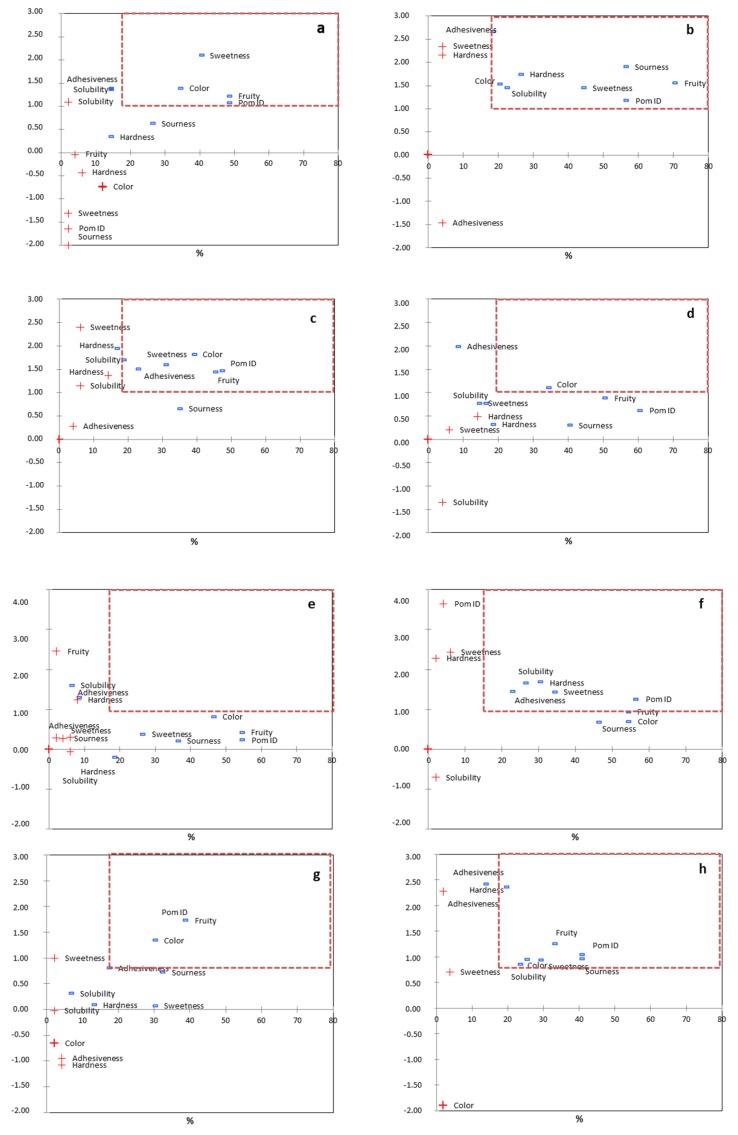
Penalty analysis of samples (sample code indicated on the top left of each figure; “too low intensity” indicated with “—”, and “too high intensity” indicated with “+”); (**a**) indicated PJAPH-20c+inf; (**b**) indicated PJAPH-20c; (**c**) indicated PJAPHS-20c+inf; (**d**) indicated PJAPHS-20c; (**e**) indicated PJH-20c+inf; (**f**)indicated PJH-20c; (**g**) indicated PJS-20c+inf; (**h**) indicated PJS-20c.

Apart from the evaluation of the liking of specific attributes, some JAR questions were also included in the affective study. For a better understanding of the relationships among JAR scores and consumer liking (with information and without information), a penalty analysis was conducted (Figure 2). The improvable attributes were those in which at least 20% of consumers caused a drop of at least 1 point for liking (critical corner). Significant differences were found for the attributes located in the critical corner among samples, with perhaps the most interesting findings being the significant effect of the information on the opinion of consumers. Among samples evaluated after providing information to consumers about formulation of jellies, all samples needed to have higher intensity of color, fruity ID, and pomegranate ID, except PJH-20c (no improvements were required). Additionally, low sweetness was detected in samples elaborated with apple purée and pomegranate juice. On the other hand, when no information was provided to consumers, the attributes to be improved in the honey- and pomegranate-based jellies (PJAPH-20c and PJH-20c, respectively) were pomegranate ID, sweetness, hardness, and solubility (all needing higher intensities).

Future studies focused on improving the fruity and pomegranate flavors and texture attributes should be optimized starting with the information reported in this study and the attributes indicated by consumers as those that needed to be improved (those located in the critical corner of Figure 2).

## 4. Conclusions

The formulation consisting of 20% gelatin with pure “*Mollar de Elche*” pomegranate juice, 1% citric acid, and sugar addition led to the best results in terms of color, texture, antioxidant capacity, and sensory attributes. The most valued quality parameter of pomegranate products, red color (a* coordinate), was not negatively affected by the process of preparing jellies. Additionally, the affective data and its relationship with descriptive sensory data showed that the main liking drivers for this specific product were: (i) high reddish color and (ii) high brightness. Application of penalty analysis showed the hardness of the jellies can be improved in at least 50% of samples assayed. It is necessary to mention the high water activity values in all samples, which will restrict the shelf life of these confections. However, further research is still needed to fully optimize this novel product; for instance, two ways of improvement can be: (i) hydrating gelatin in pomegranate juice instead of water, and (ii) optimizing the drying step. Additionally, it was clearly demonstrated how important it is to provide the consumers with the proper information. Therefore, after studying the physico-chemical, functional, and sensory parameters of the developed products, it is worth continuing this research topic and studying the effects of a blend of pectin:gelatin in the formulation of the confections, their behavior during the maturation stage and during storage, and the impact of labeling information on consumer acceptance. The development of pomegranate jelly is a good strategy to promote the consumption of pomegranate products and the present study provides useful information to understand consumer preferences and what they expect to find in these types of products.

## Figures and Tables

**Table 1:** Formulation of jelly candies consisting of pomegranate juice and apple purées.

Code *^†^*	Gelatin	Honey	Sucrose	Juice	Purée	Citric Acid
	(%) *^‡^*					
PJH-13	13	25	-	62		0
PJH-17	17	25	-	58		0
PJH-20	20	25	-	55		0
PJS-13	13	-	25	62		0
PJS-17	17	-	25	58		0
PJS-20	20	-	25	55		0
PJAPH-13	13	25	-	31	31	0
PJAPH-17	17	25	-	29	29	0
PJAPH-20	20	25	-	27.5	27.5	0
PJAPS-13	13	-	25	31	31	0
PJAPS-17	17	-	25	29	29	0
PJAPS-20	20	-	25	27.5	27.5	0
PJH-20c	20	25	-	55		1
PJS-20c	20	-	25	55		1
PJAPH-20c	20	25	-	27.5	27.5	1
PJAPS-20c	20	-	25	27.5	27.5	1

**Note:**^†^ PJ, pomegranate juice; S, sucrose; H, honey; AP, apple purée. ^‡^ The percentage of each component was expressed in weight: weight, w: w.

**Table 2:** Effect on antioxidant capacity, color coordinates, and texture properties of pomegranate jellies as affected by formulation, gelatin percentage, and type of sweetener.

Samples	Antioxidant Capacity				Color			Texture Properties *^γ^*					
	ABTS^+^	DPPH	FRAP	TPC				H	A	S	Co	G	Ch
	(mg/100 g fw)				L*	a*	b*	(g)	(g s^−1^)	(g)		(g)	(g mm^−1^)
PJH-13	2.61cd	14.8a	4.51a	159a	25.1d	0.74c	3.12d	885.0d	−4790abc	0.74	0.31	269.6	209.5
PJH-17	2.51d	13.8b	3.30b	133b	23.9de	0.65cde	3.15d	1224cd	−7191.8bcde	0.55	0.31	384.8	226.9
PJH-20	2.48d	11.9de	3.57b	77.8f	22.6ef	0.49ef	3.19d	3664a	−8629cde	0.76	0.26	959.0	791.6
PJS-13	2.70bcd	11.1ef	4.59a	116cd	24.8d	1.24a	2.25ef	1542c	−2138a	0.94	0.30	467.2	444.6
PJS-17	3.64a	12.1d	4.27a	115de	25.7d	0.70cd	1.90f	820.9d	−4248abc	1.06	0.45	203.4	213.8
PJS-20	2.90bcd	14.9a	1.47f	108d	21.4f	0.68cd	2.43e	2795b	−3304ab	1.05	0.30	847.8	900.8
PJAPH-13	3.13b	12.6cd	1.86df	127bc	30.7abc	0.55de	4.19bc	791.5d	−4535abc	1.08	0.56	442.8	478.9
PJAPH-17	2.89bcd	10.8f	2.44cd	106d	29.07bc	0.13g	3.70c	1080cd	−8026cde	0.90	0.66	716.2	643.8
PJAPH-20	2.79bcd	12.1cd	1.41f	72.0f	29.6bc	0.38f	4.20b	1298cd	−9898e	1.02	0.53	692.7	704.7
PJAPS-13	3.69a	12.2cd	2.67c	92.2e	32.7a	0.47ef	5.25a	747.3d	−4309abc	1.02	0.52	386.8	393.8
PJAPS-17	3.09bc	12.9c	3.21b	159a	31.1ab	0.93b	3.76bc	836.8d	−7018bcde	0.93	0.71	595.2	551.3
PJAPS-20	2.46d	10.6f	2.04de	109d	28.9c	−0.15h	1.66f	950.5d	5662.7bcd	0.99	0.58	551.5	550.4
**Multifactor ANOVA *^†^***													
Formulation (F)	NS	**	***	NS	***	***	***	***	***	**	***	NS	NS
% Gelatin (G)	**	*	***	**	***	***	***	**	***	NS	***	NS	NS
Sweetener (Sw)	NS	NS	NS	NS	NS	***	***	NS	***	**	NS	NS	NS
F × G	**	NS	***	***	NS	NS	NS	***	NS	NS	NS	***	***
F × Sw	NS	NS	***	***	NS	NS	NS	NS	*	**	NS	NS	NS
G × Sw	NS	*	***	***	*	*	NS	***	***	NS	*	NS	
F × G × S	***	***	***	***	***	***	***	***	***	NS	NS	NS	NS
**Tukey Test *^‡^***													
F													
*PJ ^γ^*	2.80	13.1a	3.62a	118	23.9b	0.75a	2.68b	16345a	−5180a	0.84 b	0.32 b	478.4	418.9
*PJAP*	3.01	11.9b	2.27b	111	30.3a	0.38b	3.82a	642.6b	−6643b	1.00a	0.57a	372.7	372.4
G													
13%	4.01a	12.7a	4.13a	145a	28.3a	0.75a	3.70a	991.6b	−3943a	0.94	0.42 b	391.6	381.7
17%	3.60ab	12.2ab	3.26b	129ab	27.4a	0.60b	3.13b	787.0b	−6462b	0.86	0.50 a	323.0	275.8
20%	2.81b	11.5b	2.43b	107b	25.6b	0.35c	2.92b	1638a	−7320b	0.94	0.42 b	561.9	526.4
S													
Honey	3.42	12.2	3.55	122	26.8	0.49b	3.59a	1223	−7249b	0.84b	0.42	403.5	384.7
Sucrose	3.53	12.1	2.99	133	27.4	0.65a	2.91b	1054	−4568a	1.00a	0.47	447.6	406.6

**Note:**^†^ NS = not significant at *p* < 0.05; *, **, and ***, significant at *p* < 0.05, 0.01, and 0.001, respectively. ^‡^ Values (mean of 3 replications) followed by the same letter, within the same column, were not significantly different (*p* < 0.05), according to Tukey’s least significant difference test. ^γ^ Texture Properties: H, Hardness; A, Adhesiveness; S, Springiness; Co, Cohesiveness; G, gumminess; Ch, Chewiness; PJ, pomegranate juice; S, sucrose; H, honey; AP, apple purée.

**Table 3:** Effect on color coordinates, antioxidant capacity, and total polyphenol content of pomegranate jellies as affected by formulation, gelatin percentage, and type of sweetener.

Samples	L*	a*	b*	ABTS^+^	DPPH	FRAP	TPC
				(mg/100 g fw)			
PJH-20	23.9bc	0.65c	3.15b	2.51c	13.8ab	3.30b	74.1d
PJS-20	25.7b	0.70c	1.90c	3.34b	12.1c	4.27a	124bc
PJAPH-20	29.1a	0.13d	3.70b	2.99bc	10.8d	2.44bc	107c
PJAPS-20	31.1a	0.93bc	3.76b	3.09bc	12.9bc	3.21b	139ab
PJH-20c	19.7d	0.85bc	1.72cd	2.45c	13.9ab	1.18d	133abc
PJS-20c	23.2c	1.94a	0.98d	3.68b	13.6ab	3.31b	115bc
PJAPH-20c	29.6a	1.06b	6.24a	4.83a	14.4a	2.81bc	107c
PJAPS-20c	28.7a	0.17d	5.82a	2.46c	12.5c	1.17d	160a
**Multifactor ANOVA *^†^***							
Formulation (F)	***	***	***	NS	NS	NS	NS
Citric acid (C)	***	***	***	NS	***	***	*
Sweetener (Sw)	***	***	**	NS	NS	NS	*
F × C	*	NS	***	NS	NS	NS	NS
F × Sw	*	NS	***	***	NS	***	NS
C × Sw	NS	NS	NS	***	NS	NS	NS
F × C × Sw	***	***	*	*	***	**	***
**Tukey Test *^‡^***							
*F*							
PJA *^γ^*	23.1b	1.05a	1.95b	3.07	13.3	3.02	112
PJAP	29.6a	0.58b	4.89a	3.32	12.6	2.41	128
C							
0%	27.4a	0.61b	3.14b	3.03	12.4b	3.31a	111b
1%	25.3b	1.02a	3.70a	3.35	13.6a	2.11b	129a
Sw							
H	25.6b	0.69b	3.13b	3.17	13.2	2.43	105b
S	27.2a	0.94a	3.72a	3.22	12.8	2.99	134a

**Note:**^†^ NS = not significant at *p* < 0.05; *, **, and ***, significant at *p* < 0.05, 0.01, and 0.001, respectively. ^‡^ Values (mean of 3 replications) followed by the same letter, within the same column, were not significantly different (*p* < 0.05), according to Tukey’s least significant difference test. ^γ^ PJ, pomegranate juice; S, sucrose; H, honey; AP, apple purée.

**Table 4:** Variation of formulation and sweetener on descriptive sensory attributes of pomegranate jelly candies (PJH, PJS, PJAPH, PJAPS).

Samples	Color	Brightness	Sweetness	Sourness	Fruity	Pomegranate ID	Honey ID	Apple ID	Solubility	Hardness
PJH-20c	0.6b	8.8a	1.8b	0.9	2.3ab	0.8	0.2b	1.8a	5.7	3.4
PJS-20c	4.0a	8.4a	1.5b	0.4	1.9b	0.3	0.3b	0.3b	6.3	2.9
PJAPH-20c	0.1b	6.3b	2.1ab	0.4	2.2ab	0.5	0.4b	1.5a	5.9	3.1
PJAPS-20c	0.1b	6.3b	2.7a	0.8	2.7a	0.8	1.4a	1.6a	5.0	3.3
**ANOVA Multifactor *^†^***										
Formulation (F)	***	***	*	NS	NS	NS	**	*	*	NS
Sweetener (Sw)	***	NS	NS	NS	NS	NS	**	**	NS	NS
F × Sw	***	***	***	NS	*	NS	**	**	**	NS
**Tukey Test *^‡^***										
F										
PJc *^γ^*	2.3a	8.6a	1.6b	0.7	2.1	0.5	0.2b	1.0b	6.0a	3.2
PJAPc	0.1b	6.3b	2.4a	0.6	2.4	0.6	0.9a	1.5a	5.5b	3.2
Sw										
Honey	0.3b	7.5	1.9	0.7	2.2	0.6	0.3b	1.6a	5.8	3.3
Sucrose	2.0a	7.4	2.1	0.6	2.3	0.5	0.8a	0.9b	5.7	3.1

**Note: ^†^** NS = not significant at *p* < 0.05; *, **, and ***, significant at *p* < 0.05, 0.01, and 0.001, respectively. ^‡^ Values (mean of 3 replications) followed by the same letter, within the same column, were not significantly different (*p* < 0.05), according to Tukey’s least significant difference test. ^γ^ PJ, pomegranate juice; S, sucrose; H, honey; AP, apple purée.

**Table 5:** Mean scores and ANOVA for color, appearance, flavor notes, basic taste, texture, and overall liking for consumers.

Samples	Overall	Color	Appearance	Fruity ID	Pom ID	Sweetness	Sourness	Hardness	Solubility	Adhesiveness
PJH-20c	4.8b	4.4b	5.0c	4.4b	4.3	5.2	5.0	5.1	5.7	5.5
PJS-20c	5.9a	6.1a	6.2ab	5.1ab	4.7	5.2	4.8	5.5	5.2	5.7
PJAPH-20c	4.8b	4.4b	5.0c	4.4B	4.3	5.2	5.0	5.1	5.7	5.5
PJAPS-20c	5.0ab	4.6b	5.3bc	4.6ab	4.3	5.1	5.0	5.2	5.5	5.5
PJH-20c+inf	5.2ab	4.7b	5.5abc	4.5b	4.4	5.1	5.1	5.5	5.7	5.9
PJS-20c+inf	6.0a	6.0a	6.5a	5.5a	5.0	5.7	5.5	6.0	6.0	6.0
PJAPH-20c+inf	5.2ab	4.7b	5.5abc	4.5b	4.4	5.1	5.1	5.5	5.7	5.9
PJAPS-20c+inf	5.5ab	5.1ab	6.0ab	4.9ab	4.9	5.2	5.4	5.4	5.7	5.8
**ANOVA Multifactor *^†^***										
Formulation (F)	NS	NS	NS	NS	NS	NS	NS	NS	NS	NS
Sweetener (Sw)	*	***	**	**	NS	NS	NS	NS	NS	NS
Information (Inf)	*	NS	**	NS	*	NS	***	*	**	*
F × Sw	***	***	***	**	NS	NS	NS	NS	NS	NS
F × Inf	NS	NS	NS	NS	NS	NS	NS	NS	NS	NS
Sw × Inf	NS	NS	NS	NS	NS	NS	NS	NS	NS	NS
F × Sw × Inf	***	***	***	**	NS	NS	NS	NS	NS	NS
**Tukey Test *^‡^***										
F										
PJc *^γ^*	5.5	5.3	5.8	4.9	4.6	5.3	5.1	5.5	5.6	5.8
PJAPc	5.4	5.1	5.7	4.8	4.6	5.0	5.2	5.3	5.4	5.6
Sw										
H	5.2b	4.9b	5.6b	4.6b	4.5	5.1	5.1	5.3	5.5	5.7
S	5.6a	5.6a	6.0a	5.1a	4.7	5.3	5.2	5.5	5.5	5.7
Inf										
Yes	5.6a	5.3	6.0a	4.9	4.8a	5.3	5.4a	5.6a	5.8a	5.9a
No	5.2b	5.2	5.5b	4.7	4.4b	5.1	4.8b	5.2b	5.3b	5.5b

**Note:**^†^ NS = not significant at *p* < 0.05; *, **, and ***, significant at *p* < 0.05, 0.01, and 0.001, respectively. ^‡^ Values (mean of 3 replications) followed by the same letter, within the same column, were not significantly different (*p* < 0.05), according to Tukey’s least significant difference test. ^γ^ PJ, pomegranate juice; S, sucrose; H, honey; AP, apple purée.
